# Assessment of safe injection awareness and practices among healthcare providers at primary health care facilities

**DOI:** 10.1186/s42506-022-00123-3

**Published:** 2023-01-05

**Authors:** Lamia Ali, Randa Eldessouki

**Affiliations:** Department of Public Health and Community Medicine, Faculty of Medicine, Fayoum University, Faiyum, Egypt

## Abstract

**Background:**

Unsafe injection necessitates some preventive steps including promoting and assuring the execution of safe injection administration and waste disposal methods. The present study aimed to assess the awareness and practices of safe injection among health care providers working at all primary health care (PHC) facilities in Fayoum governorate, Egypt. Also, it assessed the awareness, readiness, and response related to needle stick injuries (NSIs).

**Methods:**

A cross-sectional observational study conducted from September to December 2019 at all working PHC facilities in Fayoum Governorate, Egypt, resulted in enrolling 685 health care providers, and observation of 520 injection processes. Data were collected by a combination of staff interviews and structured observation of different injection processes using the WHO revised C tool.

**Results:**

Safe injection and post-exposure NSI policies and procedures was implemented in 96.5% of the PHC facilities. Compliance to hand wash before preparing a procedure was 56.7%. Immediate disposal of used needles was 76.2% in observed injections. Hepatitis B vaccination rate among participants was 87.2%. Most participants 87.6% admitted the existence of a NSI reporting system but only 38.8% of those who had experienced NSI event reported. The rate of NSI was 14%.

**Conclusions:**

Fayoum PHC facilities have good awareness level among providers and broadly accepted compliance with injection practices as per the WHO tool. Most injection-safety aspects were satisfactory, and implemented measures to face NSI were in place. Appropriate timely actions are required to maintain the fair awareness and improve injections practices in the PHC facilities.

## Introduction

Injections are one of the world's most important health care procedures and occasionally carry the risk of transferring severe infectious diseases. The Safe Injection Global Network (SIGN) defines safe injection practices as a collection of strategies aimed to improve safe patient injection behavior without endangering the health care provider or the entire community [1].

Unsafe injection practices are prevalent in a wide range of healthcare institutions in developing countries and are involved in a variety of preventable healthcare-related risks [2]. Although, tremendous efforts have reduced the number of hazardous injections in developing countries, but the number remains high in the WHO Eastern Mediterranean Region [3]. Root causes of the problem could be summarized into main pillars which incorporate the main risks of the problem, health care providers’ risks, and working environment risks [4].

There is a possibility that the problem could be magnified by health care providers who lack knowledge, do not receive the appropriate training, and fail to adhere strictly to the safe injection practices guidelines for handling injections or their disposal. These drawbacks, in turn, lead indirectly to an increase in the blood borne transmitted infections to the providers by needle pricks or to the patients and magnify the burden of combating them [3].

The unsuitable working place and circumstances, as lack of supplies and related equipment and disposal items, have an obvious effect on hazardous unsafe injections. Unavailability of waste management protocols ensuring safety for all or lack of satisfied adherence to them are considered a hazardous risk to the whole community. In addition, the irregularity of monitoring the practice and shortage in control measures play also a part [1, 4].

The safe injection practices implemented by any health care organizations including primary health care (PHC) ones, shall pose neither harm to the patient nor the provider or the whole community [3]. Ensuring the safe appropriate administration of an injection by a well-trained safe professional healthcare provider by a sterile device (needle, syringe, etc.) and its proper disposal in a puncture-proof sharps disposal container at well-equipped and protected environment is the rational preventative measures to be achieved by any medical facility [1, 5].

Few published studies evaluated the implemented safe injection practices comprehensively including health care providers and working environment especially at primary health care facilities [2]. The aim of this study was to assess the awareness and practices of safe injection among health care providers working at primary health care facilities in Fayoum Governorate, as well as the level of awareness, preparedness, and response to needle stick injuries (NSIs).

## Methods

### Study design and location

This cross-sectional study was conducted in Fayoum, one of Upper Egypt’s governorates. Fayoum is divided into seven districts, one urban and six rural, with a total of 197 primary health care facilities (173 of which were operational) [6]. The research was conducted throughout September and December 2019 during weekday working hours.

### Subjects

The participants were health care providers, including physicians and nurses employed by the Ministry of Health in Fayoum PHC facilities. They were involved with any skin piercing by a syringe and/or needle to prescribe and/or administer a curative substance, vaccine, or family planning injections to a patient. Additionally, laboratory technicians who provide patients with phlebotomy services were included. Intradermal injections were excluded in accordance with the WHO tool.

Participants were randomly chosen from family planning, vaccination, and outpatient clinics, as well as laboratory units, at each facility. Each participant provided informed consent prior to an interview and/or observation. Patients’ rights and privacy were not violated as verbal agreement was obtained from each involved patient prior the start of the observation. Patients in the current study were not part of the participants. Only the process of care delivered to them was the observant focus.

### Sample size sampling technique

Epi Info version 7 was used to calculate the sample size under the following assumptions: Awareness and practice levels of 50%, the precision of 5%, and confidence interval of 95%; 341 was the initial number then it was doubled and increased by 10% to account for non-responses. The study achieved 91.3% response rate.

A multi-stage stratified random cluster sampling approach was established, the governate is divided into 7 health districts, each of them is considered as a stratum. In each stratum, all operating PHC facilities were visited. The cluster referred to the categories of health care providers in each stratum, 685 participants from all operating primary health care facilities were recruited to fit the predetermined sample size.

In each visited facility, participants were enlisted according to their categories and chosen through a systematic random sampling technique of the workers attending the visit the day of collecting data. Four participants were selected and interviewed from each facility after obtaining the consent.

Regarding the injection processes observed, at the visit of each institution, a random choice of at least three injection processes was observed as indicated in the WHO Tool. Each observed injection process was treated separately apart from the participants’ interview and data were filled using the observation checklist items. Valid 520 injection processes were included.

### Data collection tool

The valid and reliable questionnaire from the revised WHO Tool C for the evaluation of unsafe practices was used to collect data by a combination of staff interviews and structured observation of different injection processes [7].

1-Interviews with healthcare professionals were conducted using a questionnaire. It comprised demographic information about the participants and twelve items that assessed their awareness of general safe injection items, disposal, and supply. Awareness, readiness, and response items about needle stick injury (NSIs) were also included. Questions with correct answers scored one point, while questions with incorrect answers scored zero points. The entire score was divided into three categories: poor awareness at ≤ 50% (0–5), accepted awareness at 51–74.9% (6–8), and good awareness at ≥ 75% (9–12).

2-A checklist was designed to document every observed injection procedure. Additionally, it includes the occupations of observed health care providers, as well as the types and methods of injections observed. Twenty observational items were used to measure participants' practice of safe injection. The overall score for this group of elements was between 0 and 20. Correct participant practice earned one point, whereas observations of wrong participant practice earned zero points. The overall score was divided into three categories: poor (0–10), acceptable (11–14), and good (15–20). Some statements were phrased so that their scores were reversed to align with other items on the same scale.

### Statistical analysis

Collected data were analyzed using the Statistical Package for Social Science (SPSS) version 25. The Mean and SD were calculated for quantitative variables, while numbers and percentages were computed for descriptive ones. Chi-square, independent *t* test, or one-way ANOVA were used as tests of significance. Pearson correlation and multiple regression analysis were also used to assess the correlation between different variables. A *P* value of ≤ 0.05 was considered statistically significant.

## Results

The study enrolled 685 health care employees in various positions from PHC facilities. Nurses were the prevalent category of participants 53.2%, followed by physicians 29.8% then technicians 17.1%. Fifty percent of participants were between the ages of 35 and 54, and 71.2% were females compared to 28.8% males. Most of participants (70.2%) were involved in the injection process, whereas 84.7% had received all three doses of the hepatitis B vaccine.

The highest percentage of all categories of participants (96.4 %) knew implemented safe injection rules and procedures and implemented rules and procedures for post-exposure to NSI (Table 1).

**Table 1:** Awareness of participants of different work positions about safety injection practices at Fayoum’s primary health care facilities, 2019

Awareness items about safety injection practices (correct answers displayed)	Work position				
	Physicians ***N*** (%)	Nurses ***N*** (%)	Phlebotomists ***N*** (%)	Total sample ***N*** (%)	***P*** value
There are implemented safe injection policy and procedure in the center	196 (96.1)	348 (95.6)	117 (100)	661(96.5)	0.07
Some diseases could be transmitted through contaminated devices (unsafe injection practice)	204(100)	364(100)	117(100)	685(100)	-
**Awareness about needle stick injury (NSI) exposure items**					
There are implemented policies and procedures for post-exposure of NSI	196 (96.1)	348 (95.6)	117 (100)	661(96.5)	0.07
Knowing measures to be taken in case of NSI exposure	176(86.3)	304(83.5)	105(89.7)	585(85.4)	0.12
There is implemented reporting system after NSI exposure	178(87.3)	308 (84.6)	106 (90.6)	592(86.4)	0.21
**Awareness about supply items**					
Adequate stocks of syringes during last 6 months	102(50)	277(76.1)	**94(80.3)**	473(69.1)	.001*
Regular supply of sharp boxes during last 6 months	47(23)	**238(65.4)**	87(12.7)	372(54.3)	.001*
**Awareness about disposal items**					
There are health care waste disposal policy/guidelines or similar by the Ministry	53(26)	276(75.8)	**93(79.5)**	422(61.6)	.001*
There are designated staff for disposing health care waste	109(53.4)	283 (77.7)	**94(80)**	486(70.9)	.001*
N-recapping of syringes	146(76.5)	326(89.6)	**108(92.3)**	590(86.1)	.001*
Immediate disposal of used syringes in sharp boxes	144(70.6)	327(89.8)	**106(90.6)**	577(84.2)	.001*
N-disposing sharp boxes when being full	122(59.8)	300(82.4)	**99(84.6)**	521(76.1)	.001*

*N* items that are negatively worded and the correct response is NO

*P value ≤ 0.05 is considered significant

Around 80% of technicians (80.3%) reported having enough syringe stocks in the last 6 months whereas 65.4% of nurses reported having a regular supply of sharps containers in the last 6 months. The differences in these two items among different work positions were statistically significant (*p* = 0.001) (Table 1).

Overall, 422 of participants (60.6%) were aware of the existence of waste disposal policies/guidelines, and 70.9% reported that there is designated staff for disposing of health care waste. Most participants (86.1%) were aware that reusing needles is not recommended, whereas 84.2% were aware that immediately disposing used syringes in sharp boxes is the proper practice. However, only 76.1% of participants were aware that it is not suggested to dispose sharp containers when they are full. There were statistically significant differences between these questions across all participant categories (*p* = 0.001); technicians showed the highest levels of awareness, while physicians demonstrated the lowest levels of awareness, as shown in (Table 1).

Five hundred twenty injection processes were observed; 62.1% of them were vaccination, followed by lab investigations (21.1%), then family planning (3.5%). Just 3.5% of studied processes involved therapeutic injections.

Only 56.7% of observed providers washed their hands before the injection was prepared. Only 45.1% of providers used new gloves, with a statistically significant difference in favor of laboratory investigations across different working groups (*p* = 0.001), as seen in Table 2. Only 32.5% of patients brought their needles and syringes, with the highest percentage, 89.1%, bringing them for lab investigations and none bringing them for vaccinations. There were statistically significant differences among working groups (*p* = 0.001) (Table 2).

**Table 2:** Injection process practices observed among observed staff who had injection role at Fayoum’s primary health care facilities, 2019

Injection process practices observed	Type of injection					
	Lab investigation ***N*** (%)	Vaccination ***N*** (%)	Family planning ***N*** (%)	Therapeutic injection ***N*** (%)	Total sample ***N*** (%)	***P*** value
The provider cleaned his/her hands before preparing an injection (either water and soap or alcohol based hand rub (*n* = 520)	70(63.6)	174(53.9)	41(59.4)	10(55.6)	295(56.7)	0.33
The provider wore a new pair of gloves, *n* = 520	78(70.9)	120(37.2)	26(37.1)	11(61.1)	235(45.1)	.001*
N-patient brought his/her syringe and needle for the observed injection *n* = 520	98(89.1)	0(0)	59(85.5)	12(66.7)	169(32.5)	**.001**
Injections were prepared by aseptic technique in clean area *n* = 410	NA	323(100)	69(100)	18(100)	410(100)	–
The provider used a new syringe every time before procedure, *n* = 520	110(100)	323(100)	70(100)	18(100)	520(100)	–
Needle and syringe were used for only one patient	110(100)	323(100)	70(100)	18(100)	520(100)	–
The rubber cap on medication vial is disinfected with alcohol rub prior piercing *n* = 410	NA	190(58.5)	43(62.3)	10(55.6)	243(59.2)	0.07
Multi-dose vials were entered every time with a new needle and syringes *n* =323	NA	323(100)	NA	NA	323(100)	–
Multi-dose vials were dated when they had been first opened and discarded within 28 days unless there are some manufactured precautions, *n* = 323, *p* value	NA	260(80.5)	NA	NA	260(80.5)	–
A gauze barrier was used when breaking glass ampoules to avoid stick injuries *n* = 410	NA	169(52.3)	28(40)	16(88.9)	213(51.9)	.001*
Readymade sterile water or manufacture’s after for reconstitution of vials was use, *n* = 410	NA	287(88.9)	60(85.7)	12(66.7)	359(87.5)	.001*
The expire and validity of drugs exposure to heat or light was checked, *n* = 410	NA	260(80.5)	56(80)	12(66.7)	328(80)	.001*
The injection site was cleansed and/or disinfected before the injection, *n* = 520	78(70.9)	233(72.1)	50(71.2)	10(55.6)	371(71.2)	0.51
Aseptic non-touch technique for the vein was applied after preparation, *n* = 128	110(100)	NA	NA	18(100)	128(100)	–
After the procedure, the provider used a clean gauze pad and gently apply pressure to the puncture site to stop bleeding, *n* = 520	78(70.9)	152 (47.1)	35(50)	12(66.7)	277(53.2)	.001*
N-recapping of syringes was done by the provider *n* = 520	80(72.7)	248(76.8)	51(72.9)	6(33.3)	385(73.9)	.001*
After procedure, a needle remover or needle destroyer was used for each procedure, *n* = 520	0(0)	2(.6)	0(0)	0(0)	2(.6)	0.71
Immediate disposal of used needles and syringes in sharp box/container was done, *n* = 520	52(47.3)	273(84.5)	60(85.7)	12(66.7)	397(76.2)	.001*
After procedure, the provider cleaned the work area with disinfectant if there is blood or body fluid contamination, *n* = 520	96(87.3)	177(54.8)	41(58.6)	16(88.9)	330(63.3)	.001*
After procedure, the provider cleaned his/her hands (either water and soap or alcohol based hand rub), *n* = 520	86(78.2)	225(69.7)	50(71.4)	12(66.7)	373(71.6)	0.31

N items that are negatively worded and the correct response is NO

*P value ≤ 0.05 is considered significant

In 87.5% of observed injections, ready-made sterile water or manufacturer’s water was used to reconstitute vials, with a statistically significant difference among groups in favor of vaccination (*p* = 0.001). Regarding the use of gauze to prevent stick injuries when breaking glass ampoules, it was practiced by only 51.9% with a statistically significant difference among groups in favor of therapeutic injection (*p* = 0.001). The expiration and validity of drugs exposed to heat or light were verified in 80% of observed injections, with therapeutic injections having the lowest rate.

Immediate disposal of used needles was observed in 76.2% of observed injections, with statistical significance in favor of family planning injections (85.7%) and 47.3% of laboratory investigation procedures. Significant differences were observed among groups (*p* = 0.001).

Avoiding syringe recapping was statistically significantly different among working groups with the lowest rate of therapeutic infection. In contrast, the significant difference among groups was in favor of laboratory investigations when it came to utilizing clean gauze to apply pressure to stop bleeding (Table 2).

After categorizing awareness into three levels, we conducted an additional analysis. It shows that a high level of awareness (≥ 75%) predominated all other levels of awareness. There were highly significant differences between participants’ work positions (*p* = 0.001). Around 75% of laboratory technicians were classified as having a good awareness level, followed by 63.7% of nurses and 23% of physicians.

In terms of practice level, the accepted level (51–74.9%) accounted for 62.3% and dominated all other practice levels (Table 3).

**Table 3:** Comparison of awareness level and injection process practice level among participants according to work position at Fayoum’s primary health care facilities, 2019

Level of awareness and injection process practice	Work position NO (%)				
**Awareness level**	**Physicians** ***N*** **= 204**	**Nurses** ***N*** **= 364**	**Laboratory technicians** ***N*** **= 117**	**Total** ***N*** **= 685**	***P*** **value**
Poor awareness ≤ 50% (0–5)	76 (37.3)	30(8.2)	1(0.9)	107 (15.6)	.001*
Accepted awareness level 51–74.9% (6–8)	81(39.7)	102(28)	29(24.8)	212(30.9)	
Good awareness level ≥ 75% (9–12)	47(23)	232(63.7)	87(74.4)	366(53.4)	
**Injection process practice level**	**Physicians** **NA**	**Nurses** ***N*** **= 410**	**Laboratory technicians** ***N*** **= 110**	**Total** **520**	***P*** **value**
Poor practice ≤ 50% (0–10)	NA	74(18)	36 (32.7)	110(21.2)	.001*
Accepted practice level 51–74.9 % (11–14)	NA	250(61)	74(67.3)	324(62.3)	
Good awareness level ≥ 75% (15–20)	NA	86 (21)	0(0)	86 (16.5)	

*P value ≤ 0.05 is considered significant

A satisfactory awareness regarding needle stick injuries NSIs was observed. Awareness of the measures to be followed in case of NSIs was 85.4%, while 86.4% were aware that a reporting system is being implemented following NSIs exposure. There were no significant differences among participants based on their participation in the injection procedure, as shown in Table 4.

Regarding readiness and response towards needle stick injuries exposure, 78.2% of participants received training about NSI, and 84.7% received full doses of the hepatitis B vaccine. Of 103 (15%) participants who got exposed to NSI during the last 6 months, only 38.8% of them reported it (Table 4).

**Table 4:** Awareness, readiness, and response as regards needle stick injuries exposure among participants according to their role in injection process at Fayoum’s primary health care facilities, 2019

Needle stick injuries exposure items and the readiness to deal with them	Participants had role in injection process		Total (685)	***P*** value
	Yes (481)	No (204)		
**Awareness about needle stick injury (NSI) exposure items**				
There are implemented policies and procedures for post-exposure of NSI	465(96.7)	196 (96.1)	661(96.5)	0.41
Knowing measures to be taken in case of needle stick injury (NSI)	409(85)	176(86.3)	585(85.4)	0.72
There is implemented reporting system after NSI exposure	409(85)	178(87.3)	592(86.4)	0.21
**Readiness and response to needle stick injury (NSI)**				
Received training about needle stick injury (NSI) policy	380(76.5)	156(79)	536(78.2)	0.72
In last 6 months, there was at least one exposure to needle stick injury (NSI)	62 (30.4)	41(8.5)	103(15)	.001*
Post-exposure prophylactic medications are provided after Needle stick injury (NSI)	260(54.1)	101(49.5)	361(52.7)	0.42
Implemented reporting system for NSI	419(87.1)	181(88.7)	600(87.6)	0.71
Experienced NSI were reported, *n* = 103	35(33.9)	5(4.8)	40(38.8)	0.04*
Received full doses of hepatitis B vaccine	407(84.6)	173 (84.8)	580(84.7)	0.52

*P value ≤ 0.05 is considered significant

## Discussion

One of the core elements of primary health care services is prevention of infectious diseases and its transmission, including blood borne infection. Protection of both patients and health care providers from infection transmission at any point of care is crucial. Therefore, safe injection practices and proper infection control are basic expectations at any health care sites [8].

Our study demonstrated a good level of awareness regarding the implemented policies and procedures for safe injection and post-exposure NSI (96.5%). It represents an improvement over the percentage reported (63.2%) in a similar study in Gharbia, Egypt [9]. A reasonable explanation was the lack of many important policies and procedures for safe injection at that time in the Gharbia health care facilities.

The overall awareness level based on number of correct answers was satisfactory. However, 50% of participants had good awareness level whereas one third of participants had accepted awareness level. The lower level of accepted awareness score in comparison to the good awareness score could be attributed to the physician’s low level of awareness regarding ‘supplies’ and ‘disposal items. This could be inferred as these items were outside the scope of their direct responsibility. In contrast, lab technicians, and nurses in our findings demonstrated higher awareness levels which may be explained by their direct participation in the process of injection (supply and disposal).

Our findings were in line with similar studies which reported a wide difference in the level of awareness among different categories of health care providers. One of the main factors causing these wide variations is the level of education among participants and regions assessed as shown in a study in Beni-Suef University Hospital, Egypt, in comparison to a Saudi hospital. The participants were all nurses with post-secondary education and had attended training, the reported level of “good” knowledge was higher among Egyptian nurses [1]. Whereas a lower level was reported among health care providers across different health care facilities in North-Eastern Nigeria, who had lower level of education despite attending training [10].

The participants in our study were highly aware of essential awareness items such as disease transmission risks through contaminated equipment (100%), presence of reporting system after NSI exposure (86.4%), avoidance of recapping (86.1%), and rapid disposal of used syringes in sharp boxes (84.2%). A plausible explanation is the emphasis on these items during training and in the mainstream education of health care providers. However, these percentages were still lower than the ones reported in Oman study [3], and in Fayoum University teaching hospital study where the intervention effect of training was the cause behind the improved awareness level about safe injections [11].

By observation, full implementation was done only for four items: the use of new disposable syringe for each patient, a multi-dose vial is entered every time by a new syringe, the preparation of injection in a clean area, and the aseptic non-touch technique for the vein. In practice, other steps for safe injection were fulfilled in variable percentages. Research show that implementation of safe injection varied significantly across different types of services [12]. The vaccination procedures were the most common activities observed, which are within the scope of services provided at PHC facilities as the same locations were conducted [12, 13]. Although some patients would bring with them their own new syringe especially for lab investigations, yet this is never observed at vaccination clinic. Hence, proper management of the limited resources and the shortage is crucial.

The percentage of providers who cleaned their hands before injection preparation was higher than those reported in previous studies conducted in Egypt and Saudi Arabia [1], another at PHC centers in Alexandria, Egypt [12] and a third in Gizan [13] and in Port said general hospital [14]. Only one study conducted in West Bengal reported 100% of disinfecting hands before the procedures [15]. Better compliance was observed after the injection process but still urge the need for training and regular monitoring of practice to reach a satisfactory compliance level.

Proper disposal of the used syringe in a puncture-proof container that is closed immediately after use without recapping provide the providers with significant protection and reduce the most avoidable risk [16]. Our observation showed that the sharp box/container is the most used method for syringe disposal while the needle remover was used in only one center. More than three quarters of the used needles and syringes were properly disposed of in a sharp box/container. This percentage is lower than that reported in the comparative study conducted in Egypt and Saudi Arabia [1] but still higher than that reported by a Nigerian study [17]. The differences are mainly due to the relatively high cost of these devices for PHCs, although unsecure disposal can make it easier to scavenge, repackage, and resell.

Furthermore, although 78% participants had received training on NSI policies, 73.9% implemented no recapping. This rate is higher than some results reported in the Africa and Alexandria [12, 18] but still there is room for improvement as NSI mostly happen during recapping [19]. With the high compliance observed by the trained personnel, achieving a higher rate of participant’s training would help further reduce the percentage of NSI encountered.

In our study, 15% of participants stated that they had at least one exposure to NSIs in the previous 6 months. This percentage is similar to that reported in Jazan [12] and Alexandria [13], but lower than those reported by other studies [1, 9, 14]. This could be attributed to the higher risks of exposure in hospitals due to higher rate of injections administered. Although most needlestick injuries do not lead to infection transmission, a single incident can cause a serious chronic lifelong infection such as HIV or hepatitis to develop [20]. The risk of contracting hepatitis B is the highest of all infections [21], which necessitates full immunization of providers against Hepatitis B [22–24].

The percentage of injection providers who received full doses of hepatitis B vaccinations was similar to the one reported in a Saudi study in Jazan Region [13] and even higher than those reported by similar studies in different Egyptian regions [11, 12, 14] and in an Indian study [23]. However, full immunization of all health workers is needed and should be considered a fundamental right as Egypt has the highest rate globally for hepatitis B and C for the last 20 decades [22] with their life-long and life-threatening complications.

One of the weak points observed in safe injection practice is the low NSI reporting (38.8%) among the exposed providers for NSIs, despite the high awareness (86.4%) of the presence of an implemented NSI reporting system. It is still low and further interviews with health care providers are recommended to find out the root causes of under-reporting, which is a universal behavior by many health care personnel [13].

Overall, the comparison of awareness versus practice scores showed a statistical difference in favor of awareness. Although most health care providers are aware of safe practices, not all of them would put it into practice. This could be attributed to factors related to work such as lack of resources, work overload [2], as well as factors related to personal beliefs. Personal health beliefs are best explained by the health belief model dimensions where a lower perception of barrier and higher perception of stimuli are needed to apply knowledge into practice [19]. Reformulation of health care providers training based on the health belief model might help in achieving higher rate of knowledge translation to practice.

Furthermore, our findings showed statistically significant difference among various categories of health care providers in their awareness and practice level with the most compliant being the lab technicians though physician by education and clinical training are expected to have high level of awareness and high degree of compliance. Since our study is one of the few studies which included all the categories of health care providers and explored the differences in awareness and practice, further studies are recommended to support the findings and explore different paths for achieving optimal compliance and preventing serious sequelae.

### Study limitations

Some collected information, e.g., needlestick injuries and hepatitis B immunization, were based on self-reporting by the providers. Additionally, observation of the health workers after obtaining informed consent may affect the results towards best practices due to the Hawthorne effect. Finally, the study was conducted in one governate. Thus, other studies are recommended to identify disparities between different governates and health districts.

## Conclusions

Injections have saved many lives but at the same time carry the risk of infections. Our findings indicated a good awareness level among providers and broadly accepted compliance with injection practices as per the WHO tool. Many injection-safety aspects were satisfactory and implemented measures to face NSI were in place. However, appreciated actions are required to maintain the fair awareness of health care providers, enforce the rules, and perform regular random audits on practices for the sake of improvement. Providing hepatitis B vaccine as a compulsory measure in governmental primary health care centers is a demand in accordance with exploring and improving the underreporting causes of NSIs.

## Data Availability

The datasets generated and/or analyzed during the current study are not publicly available but are available from the corresponding author on reasonable request.

## Abbreviations

NSI: Needles stick injury
PHC: Primary health care
SD: Standard deviation
SIGN: Safe Injection Global Network
SPSS: Statistical Package for Social Science
WHO: World Health Organization
